# Electrochemical generation of phenothiazin-5-ium. A sustainable strategy for the synthesis of new bis(phenylsulfonyl)-10*H*-phenothiazine derivatives

**DOI:** 10.1038/s41598-024-53620-0

**Published:** 2024-02-21

**Authors:** Niloofar Mohamadighader, Faezeh Zivari-Moshfegh, Davood Nematollahi

**Affiliations:** Faculty of Chemistry and Petroleum Sciences, Bu-Ali-Sina University, Hamedan, 65174-38683 Iran

**Keywords:** Electrochemistry, Sustainability, Reaction mechanisms, Reactive precursors

## Abstract

In this work, the electrochemical generation of phenothiazin-5-ium (PTZ_ox_) from the direct oxidation of phenothiazine (PTZ) in a water/acetonitrile mixture using a commercial carbon anode and conventional stainless steel cathode is reported. PTZ_ox_ is a reactive intermediate with high potential synthetic applications, which is used in this paper for the synthesis of new phenothiazine derivatives. In this work a novel and simple electrochemical methodology for the synthesis of some bis(phenylsulfonyl)-10*H*-phenothiazine derivatives was established. In this paper, a mechanism for PTZ oxidation in the presence of arylsulfinic acids has been proposed based on the results obtained from voltammetric and coulometric experiments as well as spectroscopic data of the products. These syntheses are performed in a simple cell by applying constant current under mild conditions and at room temperature with high atom economy.

## Introduction

**Figure 1:**
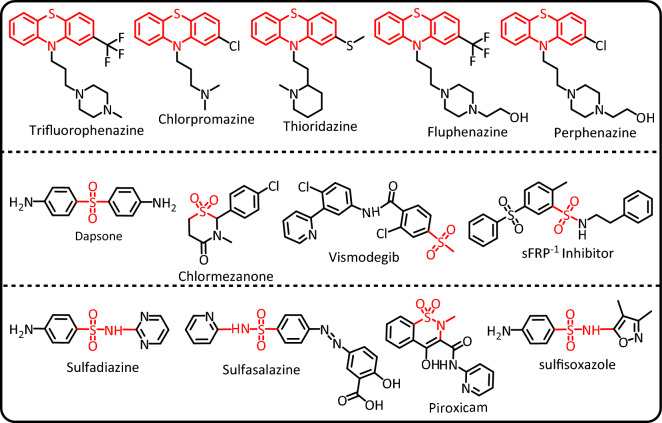
Examples of some biologically active phenothiazine, sulfone and sulfonamide drugs.

Electrochemistry is a powerful tool to identify the mechanism of reactions as well as the synthesis of organic and inorganic compounds^1–3^. In this research, we deal with three categories of compounds with valuable medicinal properties. The first category involves phenothiazines. Phenothiazine has various biological activities, such as neuroleptic, antiemetic and anti-histaminic activities, which have made it interesting to scientists^4^. Phenothiazine derivatives are also used as anti-cancer, antipyretic, anticonvulsant, analgesic, anti-fungal, anti-bacterial, anti-malarial, antiinflammatory, and immunosuppressive agents^4–6^. Prominent examples of widely used phenothiazine derivatives include trifluorophenazine, chlorpromazine, thioridazine, fluphenazine and perphenazine (Fig. 1, top row). From the electrochemical point of view, phenothiazine is a useful compound in photovoltaic^7^ and electrochemical applications^8–11^. Also due to their interesting chemical and physical properties, phenothiazines are an important and widely used building block for a wide range of applications in different fields of chemistry^12^. For example, these compounds are widely used in the optoelectronic industry due to the presence of electron-rich nitrogen and sulfur atoms in their molecules^13^. Also, since phenothiazines have a reversible redox process with low oxidation potential, they are widely used in perovskite solar cells^14^.

The second category of compounds are sulfones. Sulfone drugs are useful to treat many diseases, like anti-inflammatory^15^, antimicrobial^16^, anti-cancer^17^, and anti-malaria^18^. Diarylsulfones are useful in biological fields and pharmaceutical aims as antifungal, antimicrobial, anticancer, antibacterial and HIV treatment agents and also can be used to prepare other drugs^19,20^. Prominent examples of widely used sulfone and diarylsulfone drugs are shown in Fig. 1, middle row. The third category of compounds are sulfonamides. Sulfonamides are the antibiotics that are applied in to treat bacterial infections^21^. In addition, they are used as anti-cancer, anti-inflammatory and antiviral drugs^22–26^. Additionally, type II diabetes treatment activity is reported from some of these organic drugs^27^. Moreover various type of diseases such as coronary artery disease, asthma^28^, Alzheimer's^29^ and respiratory diseases^30^ have been treated by sulfonamide drugs which are called sulfa drugs. Examples of the most commonly used sulfonamides are shown in Fig. 1, bottom row.

The medicinal properties of these three groups of compounds prompted us to prepare new molecules containing three moieties of phenothiazine, sulfone, and sulfonamide. The presence of these three moieties in one molecule can have high potential in variable medicinal and biological properties, and maybe the synergistic effect of these groups will intensify the medicinal properties and/or reduce the side effects of the synthesized molecule. To achieve this idea, the electrochemical oxidation of phenothiazine in the presence of some arylsulfinic acids has been carried out in an undivided cell using a carbon electrode. This work presents a sustainable and efficient one-step strategy with high atom economy under ambient conditions without using any catalyst for the synthesis of novel compounds with medicinal potential.

## Experimental data

### Apparatus and reagents

**Figure 2:**
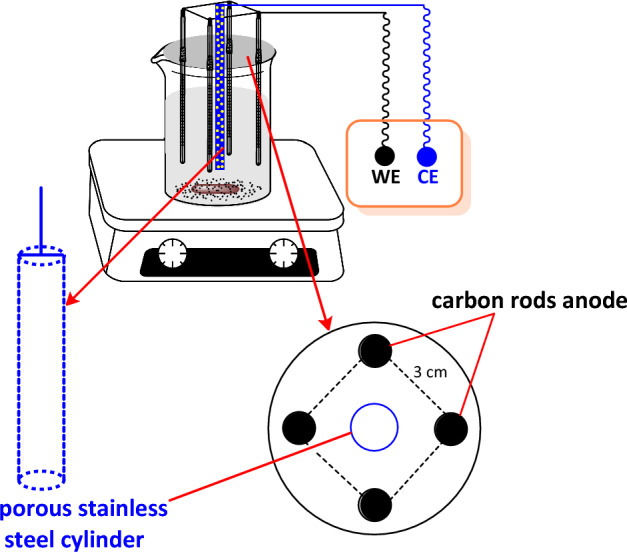
Cell used for the synthesis of phenothiazine derivatives.

Voltammetric and controlled potential coulometric experiments were carried out using an Autolab model PGSTAT20 potentiostat/galvanostat (Metrohm-Autolab, Netherland) equipped with a working electrode (glassy carbon disc, 1.8 mm diameter), a counter electrode (platinum wire) and a reference electrode (Ag/AgCl) (3M KCl). All electrodes are prepared from Azar Electrode Company (Urmia, Iran). The alumina slurry from Iran Alumina Co. (0.1–3.0 μm) was used to polish the glassy carbon electrode. The working electrode (anode) used in macroscale electrolysis consists of a set of four soft carbon rods (diameter 8 mm and length 6 cm) placed at a distance of 3 cm from each other on the edges of a square. The counter electrode (cathode) consists of a stainless steel porous cylinder (area 25 cm^2^) placed in the center of the square (Fig. 2). The electrolysis was carried out using a DC power supply model Dazheng ps-303D in an undivided cell equipped with a magnetic stirrer at room temperature. Controlled potential coulometry was performed in the same cell with an Ag/AgCl (3M KCl) reference electrode using a BEHPAJOOH-C2056 potentiostat.

Perkin-Elmer 1760 X device, German Bruker spectrometer (model: Avance 300 MHz), Agilent-5973C mass spectrometer and Barnstead Electrothermal 9100 instrument were used to record FTIR, NMR, MS spectra, and melting point, respectively. The NMR chemical shifts were related to the residual solvent signal. Phenotiazine (**PTZ**) and aryl sulfinic acid sodium salt, benzenesulfinic acid (**BSA**), 4-toluenesulfinic acid (**TSA**) and 4-chlorobenzenesulfinic acid (**CSA**) sodium salts) were obtained from Sigma-Aldrich and used without further purification. The buffer solution with pH value of 2.0 was prepared by 0.2 M phosphoric acid (pro-analysis grade from E. Merck). The pH values were adjusted by sodium hydroxide.

### General procedure for synthesis of bis(phenylsulfonyl)-10*H*-phenothiazine derivatives (2a–2e)

The electroorganic synthesis of bis(phenylsulfonyl)-10*H*-phenothiazine derivatives (2a–2e) has been carried out under controlled potential as well as constant current conditions at room temperature. In controlled potential electrolysis **PTZ** (0.25 mmol) and arylsulfinic acid (0.5 mmol) were electrolyzed at 0.55 V versus Ag/AgCl (3M KCl) in the mixture of water (phosphate buffer, pH 2.0, *c* = 0.2 M)/acetonitrile (50/50, v/v) (80 ml). The progress of the electrolysis was monitored by periodically recording the decrease of the oxidation peak current in cyclic voltammetry and also by using TLC on silica gel (ethyl acetate/*n*-hexane: 40/60). The samples on the TLC were visualized with a UV lamp (254 nm). To activate the electrode surface, the electrolysis is stopped for a while and the carbon anodes are washed with acetone. Electrolysis was terminated when the oxidation peak current reached 5% of the initial value. In this condition, the amount of electricity consumed is equal to 100 C. At the end of the electrolysis, the contents were placed at room temperature to reduce the volume of the solution (evaporation) to half and lead to the precipitation of the products. The crude precipitate was filtered and washed several times with water. After drying the product was purified by thin layer chromatography on silica gel GF250-60 with ethyl acetate/*n*-hexane (40/60 V/V). Product yield is calculated by weighing the pure product.

Electrolysis under constant current conditions has also been performed by applying a current density of 1.25 mA/cm^2^ (30 mA) for 78 min (consumption of 140 C electricity) for oxidation of **PTZ** in the presence of **BSA** as well as other nucleophiles (**TSA** and **CSA**) under similar conditions as reported for electrolysis in controlled potential method.

### Characteristics of the products


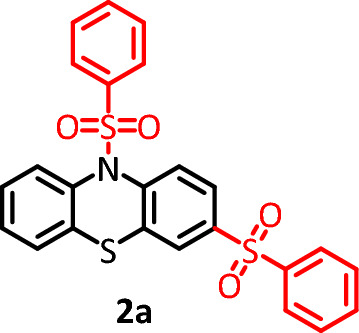


#### 3,10-bis(phenylsulfonyl)-10H-phenothiazine (C_24_H_19_NO_4_S_3_) (2a)

Yellow powder (47.5 mg), isolated yield: 39%. Mp: 153–156 °C: ^1^H NMR, *δ* ppm (300 MHz, DMSO-*d*_6_), 6.2 (s, 1H, aromatic), 6.55 (d, *J* = 3.9 Hz, 2H, aromatic), 6.67–7.06 (m, 4H, aromatic), 7.49 (m, 7H, aromatic), 7.90 (d, *J* = 4.8 Hz, 3H, aromatic). ^13^C NMR, *δ* ppm (75 MHz, DMSO-*d*_6_): 114.7, 115.9, 118.0, 123.6, 127.4, 127.6, 128.8, 129.7, 130.0, 130.2, 132.8, 133.7, 134.0, 134.4, 140.3, 142.4, 147.0. IR (KBr) (cm^−1^): 2958, 2921, 2851, 1731, 1603, 1563, 1470, 1446, 1384, 1320, 1153, 1111, 1075, 722. MS (m/z) (EI, 70 EV) (relative intensity): 77 (30), 154 (40), 198 (90), 339 (100), 396 (20), 479 (M, 30).
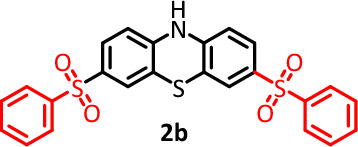


#### 3,7-bis(phenylsulfonyl)-10H-phenothiazine (C_24_H_19_NO_4_S_3_) (2b)

Cream powder (60.0 mg), isolated yield: 50%. Mp: 120–123 °C. ^1^H NMR, *δ* ppm (300 MHz, DMSO-*d*_6_), 6.76 (d, *J* = 8.4 Hz, 2H, aromatic), 7.46 (d, *J* = 5.3 Hz, 2H, aromatic), 7.53 (d, *J* = 3.5 Hz, 1H, aromatic), 7.55 (d, *J* = 2.1 Hz, 1H, aromatic), 7.61 (d, *J* = 9.2 Hz, 3H, aromatic),7.66 (d, *J* = 6.7 Hz, 2H, aromatic), 9.72 (s, 1H, NH). ^13^C NMR,* δ* ppm (75 MHz, DMSO-*d*_6_): 115.5, 117.7, 125.8, 127.5, 128.7, 130.1, 133.9, 135.0, 142.1, 145.0. UV_max_ (EtOH): 274 nm^31^. IR (KBr) (cm^−1^): 3583, 3494, 3095, 2921, 1680, 1654, 1606, 1563, 1509, 1419, 1317, 1229, 1142, 745, 687.^31^ MS (m/z) (EI, 70 EV) (relative intensity): 77 (80), 107 (60), 291 (30), 338 (50), 396 (20), 479 (M, 100).
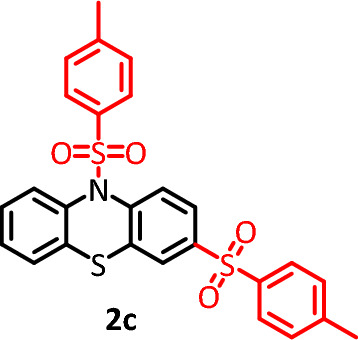


#### 3,10-ditosyl-10H-phenothiazine (C_26_H_21_NO_4_S_3_) (2c)

Yellow powder (42.5 mg), isolated yield: 34%. Mp: 160–162 °C. ^1^H NMR, *δ* ppm (300 MHz, DMSO-*d*_6_), 2.37 (s, 6H, CH_3_), 6.68–7.04 (m, 4H, aromatic), 7.39 (d,* J* = 9.0 Hz, 9H, aromatic), 7.50 (dd,* J* = 12.2 Hz, 2H, aromatic), 7.80 (d,* J* = 8.1 Hz, 3H, aromatic), 9.2 (s, 1H, aromatic). ^13^C NMR,* δ* ppm (75 MHz, DMSO-*d*_6_): 21.5, 114.6, 115.5, 115.9, 117.6, 117.9, 123.6, 125.6, 127.4, 128.4, 130.6, 133.8, 135.3, 139.2, 140.3, 144.5, 144.9, 146.8. IR (KBr) (cm^−1^): 3053, 2958, 2921, 2851, 1703, 1630, 1600, 1534, 1499, 1438, 1347, 1297,1260, 1113, 1019, 886, 770, 717. MS (m/z) (EI, 70 EV) (relative intensity): 55 (25), 91 (30), 154(30), 198 (60), 353 (100), 507 (M, 15).
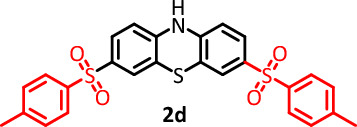


#### 3,7-ditosyl-10H-phenothiazine(C_26_H_21_NO_4_S_3_) (2d)

Cream powder (57.5 mg), isolated yield: 47%. Mp: 130–132 °C. ^1^H NMR, *δ* ppm (300 MHz, DMSO-*d*_6_), 2.37 (s, 6H, CH_3_), 6.67 (d, *J* = 6.9 Hz, 1H, aromatic), 6.72 (d, *J* = 8.4 Hz, 1H, aromatic), 6.82 (dd,* J* = 7.5 Hz, 1H, aromatic), 6.92 (d, *J* = 7.8 Hz, 1H, aromatic), 6.99 (d, *J* = 6.3 Hz, 1H, aromatic), 7.04 (d, *J* = 7.8 Hz, 1H, aromatic), 7.39 (m,* J* = 4.2 Hz, 4H, aromatic), 7.79 (dd, *J* = 10.8 Hz, 1H, aromatic), 7.78 (d, *J* = 8.1 Hz, 3H, aromatic), 9.23 (s, 1H, NH). ^13^C NMR, δ ppm (75 MHz, DMSO): 21.5, 114.6, 115.5, 115.8, 117.9, 123.6, 125.5, 126.8, 127.5, 128.2, 128.5, 130.5, 133.8, 139.6, 140.3, 144.3, 146.8. IR (KBr) (cm^−1^): 3403, 3334, 3093, 2922, 2852, 1569, 1474, 1434, 1326, 1157, 1114, 1086, 828, 745, 616, 575. MS (m/z) (EI, 70 EV) (relative intensity): 65 (15), 91 (30), 154 (40), 198 (80), 353 (100), 507 (M, 10).
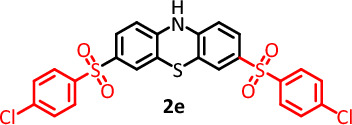


#### 3,7-bis((4-chlorophenyl) sulfonyl)-10H-phenothiazine (C_24_H_15_Cl_2_NO_4_S_3_) (2e)

Cream powder (67.5 mg), isolated yield: 50%. Mp: 145–148 °C. ^1^H NMR, (300 MHz, DMSO-*d*_6_), 6.48 (d, *J* = 7.8 Hz, 1H, aromatic), 6.54 (t, *J* = 8.4 Hz, 2H, aromatic), 6.68 (d,* J* = 6.3 Hz, 1H, aromatic), 6.77 (t, *J* = 7.8 Hz, 1H, aromatic), 7.18 (d, *J* = 2.1 Hz, 1H, aromatic), 7.29 (dd, *J* = 10.2 Hz, 2H, aromatic), 7. 43(d, *J* = 8.4 Hz, 3H, aromatic), 7.70 (d,* J* = 8.7 Hz, 2H, aromatic), 9.36 (s, 1H, NH). ^13^C NMR, δ ppm (75 MHz, DMSO): 114.7, 115.7, 115.8, 118.0, 123.6, 125.8, 126.7, 129.3, 130.2, 132.7, 138.7, 140.3, 141.4. IR (KBr) (cm^−1^): 3438, 3028, 2918, 2850, 2709, 1578, 1566, 1457, 1223, 1155, 1121, 1009, 815, 682. MS (m/z) (EI, 70 EV) (relative intensity): 51 (35), 77 (45), 158 (40), 266 (50), 407 (100), 547 (M, 10).

## Results and discussion

### Electrochemical study of PTZ in the presence of arylsulfinic acids

**Figure 3:**
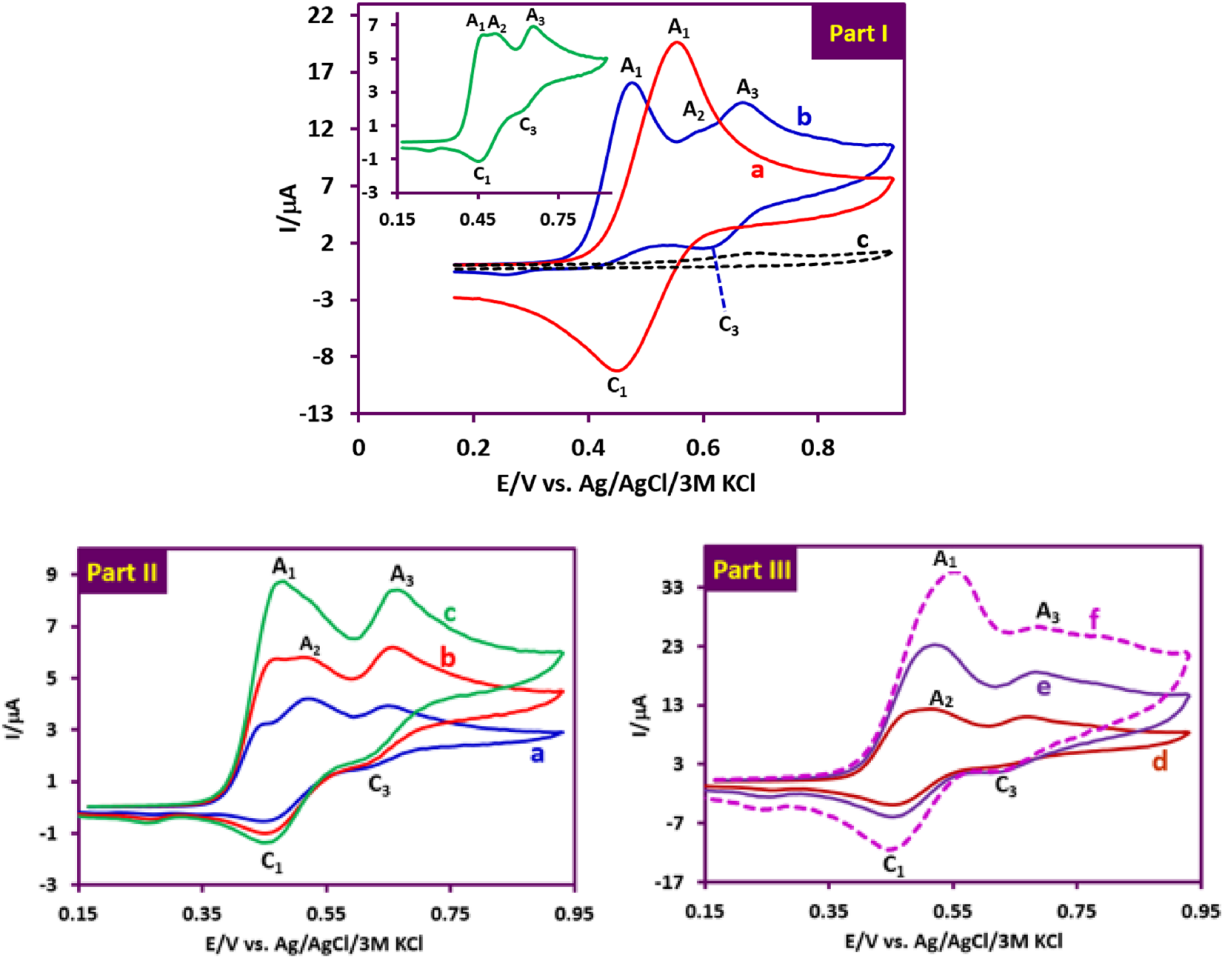
Part I: (**a**) Cyclic voltammogram of **PTZ** (1.0 mM). (**b**) Cyclic voltammogram of **PTZ** (1.0 mM) in the presence of 1.0 mM **BSA**. (**c**) Cyclic voltammogram of **BSA** (1.0 mM). Potential scan rate: 100 mV/s. inset: Cyclic voltammogram of **PTZ** (1.0 mM) in the presence of 0.5 mM **BSA**. Potential scan rate: 25 mV/s. Parts II and III: Cyclic voltammograms of **PTZ** (1.0 mM) in the presence of 0.5 mM **BSA** at different scan rates. Scan rates from a to f are: 10, 25, 50, 100, 300 and 500 mV/s. Solvent: phosphate buffer (pH 2.0, *c* = 0.2 M)/acetonitrile mixture (50/50 v/v). All experiments were performed at room temperature using a GC electrode.

The cyclic voltammogram of **PTZ** (1.0 mM) in a solution of phosphate buffer 0.2 M, pH 2.0)/acetonitrile (50/50 v/v) in scan rate of 100 mV/s is shown in Fig. 3, part I, curve a. It reveals a quasi-reversible two-electron process involving oxidation of **PTZ** to phenothiazine-5-ime (**PTZ**_**ox**_) (peak A_1_ at 0.55 V vs. Ag/AgCl) and reduction of **PTZ**_**ox**_ to **PTZ** (peak C_1_ at 0.45 V vs. Ag/AgCl)^10^. The peak current ratio (*I*_PC1_/*I*_PA1_) close to one which illustrates no side reaction is accrued in the time scale of voltammetry^10^. Figure 3, part I, curve b is the cyclic voltammogram of **PTZ** in the presence of 1.0 mM benzenesulfinic acid (**BSA**). Compared to cyclic voltammogram a, the following changes have occurred in the cyclic voltammogram of b. The first change is the removal of the cathode peak C_1_, which confirms the reaction between **PTZ**_**ox**_ and **BSA**. The second change is the appearance of an ill-defined irreversible peak A_2_ and a new reversible redox peak (A_3_ and C_3_) at more positive potentials. The peak A_2_ is related to the oxidation of the adduct formed from the reaction of **PTZ**_**ox**_ with **BSA** (**PTZ**-**BSA**) (**INT1**) to **INT1**_**ox**_ (Fig. 3, part I, curve b). The positive shift of peak A_2_ compared to peak A_1_ can be justified based on the electron withdrawing property of **BSA** bound to **PTZ**. After the electrochemical generation of **INT1**_**ox**_, another rapid chemical reaction occurs and a **BSA** binds to **INT1**_**ox**_. This chemical reaction causes the cathodic peak corresponding to the reduction of **INT1**_**ox**_ (C_2_) to be eliminated. Finally, the anodic and cathodic peaks A_3_ and C_3_ are related to the oxidation and reduction of final products (**2b**, **2d**, **2e**), respectively. The occurrence of oxidation of the final products at more positive potentials than the potential of peak A_2_ confirms the presence of two **BSA** groups in the structure of the final products. The third change is the shift of *E*_pA1_ towards less positive potentials. This is another confirmation of the reaction between electrochemically generated **PTZ**_**ox**_ and **BSA**^32^.

In Fig. 3, part I, curve c is related to **BSA** in the absence of the **PTZ**. The peak observed in this cyclic voltammogram is attributed to the one electron oxidation of **BSA** to the corresponding radical^33^. An important point regarding the peak current ratio (*I*_pC1_/*I*_pA1_) is the dependence on the nucleophile (**BSA**) concentration and on the potential scan rate. As shown in the Fig. 3, part I, inset, the peak current ratio (*I*_pC1_/*I*_pA1_) increases with decreasing **BSA** concentration. Comparison of this voltammogram with voltammogram b shows that decreasing the concentration of **BSA** slows down the reaction rate of **BSA** with **PTZ**_**ox**_ and leads to more **PTZ**_**ox**_ remaining on the electrode surface, which leads to an increase in the cathodic peak current (*I*_pC1_). The effect of scan rate on the cyclic voltammograms of **PTZ**/**BSA** mixture is shown in Fig. 3, parts II and III. As can be seen, at low scan rates such as 10 mV/s (part II, curve a), peaks A_1_, A_2_, and A_3_ are clearly observed in the anodic cycle, and peaks C_1_ and C_3_ are also observed in the cathodic scan. Increasing the potential scan rate causes remarkable two changes in the current of the peaks. The first change is related to the gradual decrease of the anodic peaks A_2_ and A_3_ with the increase of the scan rate and the second change is related to the increase of *I*_pC1_ with increasing scan rate. When the scan rate increases, there is not enough time for the **BSA** to react with **PTZ**_**ox**_. In such a situation, most of the **PTZ**_**ox**_ molecules participate in the cathodic reaction, which results in an increase in *I*_pC1_. On the one hand, the current of anodic peaks A_2_ and A_3_ decrease, because these peaks are related to the oxidation of mono-substituted and di-substituted **PTZs**, respectively, which are not formed under these conditions.

**Figure 4:**
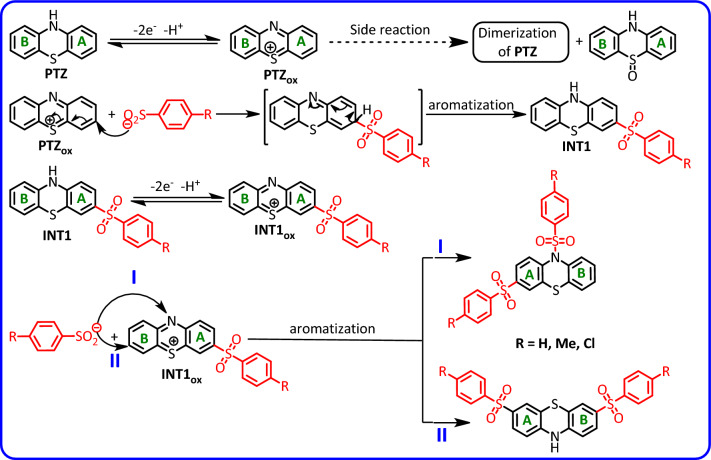
Suggested mechanism for electrochemical oxidation of **PTZ** in the presence of arylsulfinic acids.

Based on the obtained electrochemical data, the synthesis of bis(phenylsulfonyl)-10*H*-phenothiazine derivatives (**2a–e**) was carried out. The products after separation and purification were identified by various spectroscopic methods such as IR, NMR and MS. Based on spectroscopic data as well as voltammetric results, the following mechanism is proposed for the oxidation of **PTZ** in the presence of **BSA** (Fig. 4). This mechanism can be extended to other nucleophiles (4-toluenesulfinic acid, **TSA** and 4-chlorobenzenesulfinic acid, **CSA**). According to the proposed mechanism, initially **PTZ** is converted into its oxidized form (phenothiazin-5-ium, **PTZ**_**OX**_) by losing two electrons and one proton. In the next step, **PTZ**_**OX**_ is attacked by anion resulting from deprotonation of arylsulfinic acids (RSO_2_^−^) and forms the first intermediate (**INT1**) [3-(arylsulfonyl)-10*H*-phenothiazine] after aromatization. It should be noted that the formation of other isomers of **INT1** is also possible. However, due to the presence of sulfonium atom in the structure of **PTZ**_**OX**_, it is suggested that C3 atom is the most favorable site for nucleophilic attack of arylsulfinic acids.

The oxidation of **INT1** in the next step causes the formation of **INT1**_**OX**_. There are a few important points regarding this step. First, unlike **PTZ**, whose two rings A and B are similar, **INT1** rings A and B are not the same, and due to the presence of an electron-withdrawing arylsulfinic group in ring A, oxidation takes place on the B-ring. Like **PTZ**_**OX**_, **INT1**_**OX**_ is attacked by another arylsulfinic anion, leading to the final product after aromatization (Fig. 4). As seen in Fig. 4, two types of products are formed in the reaction of arylsulfinic anion with **INT1**_**OX**_. One of these products is formed by the attack of arylsulfinic anion on the nitrogen atom of **INT1**_**OX**_ (path I). This compound is a sulfone-sulfonamide product. The second product results from an attack similar to the first arylsulfinic attack (path II), which leads to the formation of the sulfone-sulfone product. These products were separated from each other by thin layer chromatography.

**Figure 5:**
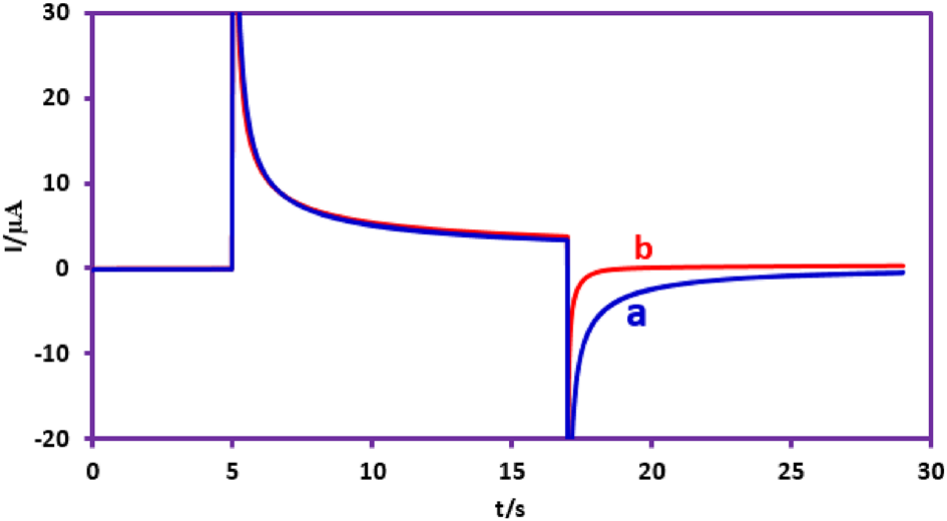
Double potential step chronoamperograms of 1.0 mM **PTZ**: (**a**) in the absence, (**b**) in the presence of 1.0 mM **BSA**. *E*_1_ = 0.20 V vs. Ag/AgCl, *E*_2_ (oxidation step) = 0.55 V vs. Ag/AgCl and *E*_3_ (reduction step) = 0.40 V vs. Ag/AgCl. Solvent: phosphate buffer (pH 2.0, *c* = 0.2 M)/acetonitrile mixture (50/50 v/v). All experiments were performed at room temperature using a GC electrode.

To further investigate the oxidation of **PTZ** in the presence of **BSA**, the double potential step chronoamperometry method was also used (Fig. 5). To achieve this goal, based on **PTZ** cyclic voltammogram (Fig. 3, part I, curve a), the electrode potential is changed from an initial value of 0.20 V to a potential of 0.55 V in the forward step (oxidation step) and then to 0.40 V in the reverse step (reduction step). The generated current was recorded for 12 s in both step. Figure 5 curve a shows the chronoamperometry of **PTZ** (1.0 mM) in phosphate buffer (pH 2.0, *c* = 0.2 M/acetonitrile mixture solution (50/50 v/v). The forward current corresponds to the oxidation of **PTZ** to **PTZ**_**ox**_ and the reverse current corresponds to the reduction of **PTZ**_**ox**_ to **PTZ**. In this method, the theoretical current ratio for a reversible system at 2*τ* and *τ* (*I*_r (2τ)_/*I*_f (τ)_) is 0.293^32,34–36^.

The experimental current ratio (*I*_r (2τ)_/*I*_f (τ)_) obtained for **PTZ** is equal to 0.25, which is slightly less than the theoretical value but close to it. This result confirms the relative stability of oxidized **PTZ** (**PTZ**_**ox**_) in the time scale of the experiment. Repeating this experiment for the oxidation of **PTZ** in the presence of **BSA** and measuring the current at 2*τ* and *τ* (*I*_r (2τ)_/*I*_f (τ)_) shows two important facts. First, the forward currents in these two experiments are exactly the same, and second, the reverse current (*I*_r (2τ)_) in the presence of **BSA** is zero. These results confirm that **PTZ**_**ox**_ is removed from the electrode surface due to its reaction with **BSA** on the time scale of the experiment.

**Figure 6:**
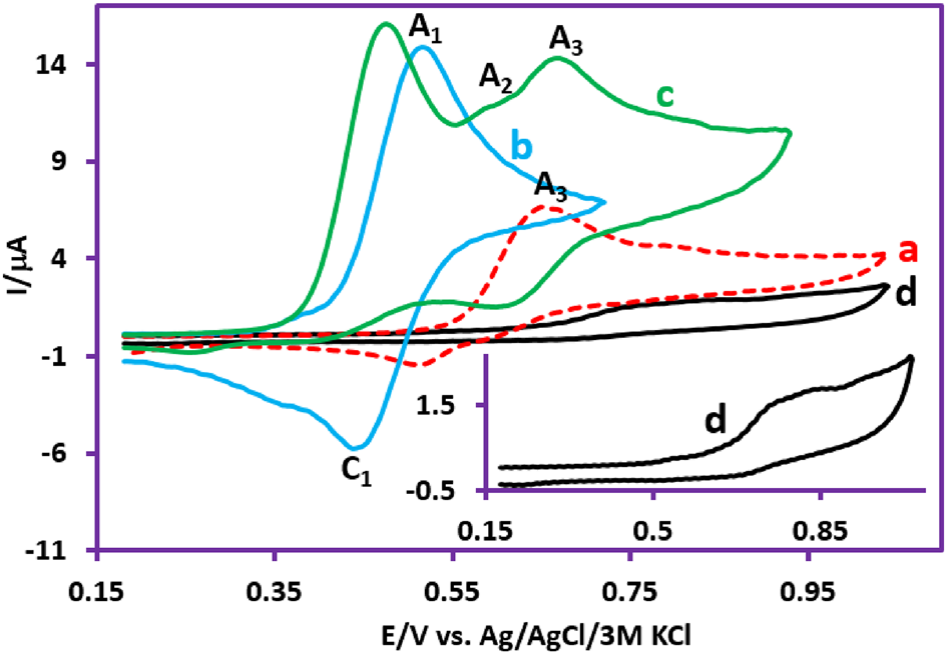
(**a**) Cyclic voltammogram of 1.0 mM **2b**. (**b**) Cyclic voltammogram of 1.0 mM **PTZ**. (**c**) Cyclic voltammogram of 1.0 mM **PTZ** in the presence of 1.0 mM **BSA**. (**d**) Cyclic voltammogram of 1.0 mM **2a**. Potential scan rate: 50 mV/s. Solvent: phosphate buffer (pH 2.0, *c* = 0.2 M)/acetonitrile mixture (50/50 v/v). Working electrode: glassy carbon electrode. All experiments were performed at room temperature.

Due to the fundamental difference in the structure of the synthesized products (3,10-bis(phenylsulfonyl)-10*H*-phenothiazine, **2a** and 3,7-bis(phenylsulfonyl)-10*H*-phenothiazine, **2b**), this section is dedicated to investigating the electrochemical behavior of the synthesized products **2a** and **2b**. Figure 6 curve a, shows the cyclic voltammogram of **2b**. The cyclic voltammogram displays a quasi-reversible redox process ascribed to the oxidation of **2b** to **2b**_**ox**_ and vice versa. Comparing the cyclic voltammogram of **2b** with that of **PTZ** (Fig. 6 curve b) shows that the oxidation potential of **2b** is about 140 mV more anodic than the **PTZ** oxidation potential. The peak shift can be expected due to the difference in the structure of **2b** (the presence of two electron withdrawing sulfonyl groups) with **PTZ**. In addition, the presence of two sulfonyl groups in the structure of **2b** has caused its oxidized form (**2b**_**ox**_) to become more unstable than the oxidized form of **PTZ** (**PTZ**_**ox**_) and thus participate in subsequent chemical reactions. As a result, the current of its cathodic peak (C_3_) becomes lower than the current of cathodic peak of **PTZ** (C_1_). Figure 6 curve d, shows the cyclic voltammogram of **2a**. In similar conditions, unlike the cyclic voltammogram of **2b**, the cyclic voltammogram of **2a** shows the presence of an irreversible process. The main reason for this difference is the attachment of a sulfonyl group to the nitrogen atom. This binding causes the two-electron oxidation of the **2a** to form a highly unstable **2a**_**ox**_ compound (Fig. 4).

**Figure 7:**
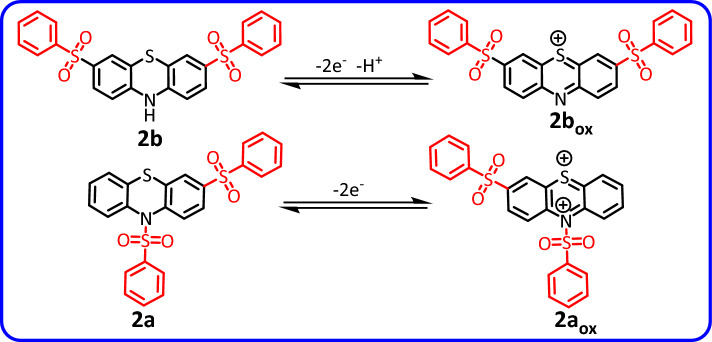
Suggested mechanism for electrochemical behavior of **2a** and **2b**.

According to the proposed mechanism in Fig. 4, the structure of the product formed via pathway II (for example **2b**) is similar to that of **PTZ** and therefore, its general electrochemical behavior should also be similar to **PTZ**. In Fig. 6, we studied and compared the cyclic voltammogram of product **2b** with the cyclic voltammogram of **PTZ**. Based on this, the redox reactions producing the anodic and cathodic peaks A_3_ and C_3_ is shown in Fig. 7. The electrochemical oxidation of **2a** was also studied (Fig. 6, curve d). Replacing the hydrogen atom in the **PTZ** molecule with a sulfinic group has a great effect on the stability of the oxidized product (**PTZ**_**OX**_). The removal of the hydrogen atom attached to the nitrogen atom as a proton and giving its electron to the mother molecule causes the relative stability of the oxidized forms of molecules **PTZ** and **2b** (**PTZ**_**OX**_ and **2b**_**ox**_). In contrast to these molecules, the replacement of the hydrogen atom with a sulfinic group in molecule **2a** has caused the oxidized molecule (**2a**_**ox**_) to be in the dication form. This compound is very unstable and quickly participates in subsequent chemical processes such as ring cleavage^37,38^, dimerization reactions^39,40^, hydroxylation reactions^41,42^, hydrolysis^43,44^ and/or sulphoxide formation^45^. The occurrence of these reactions makes the cyclic voltammogram of **2a** shows the behavior of an irreversible system.

**Figure 8:**
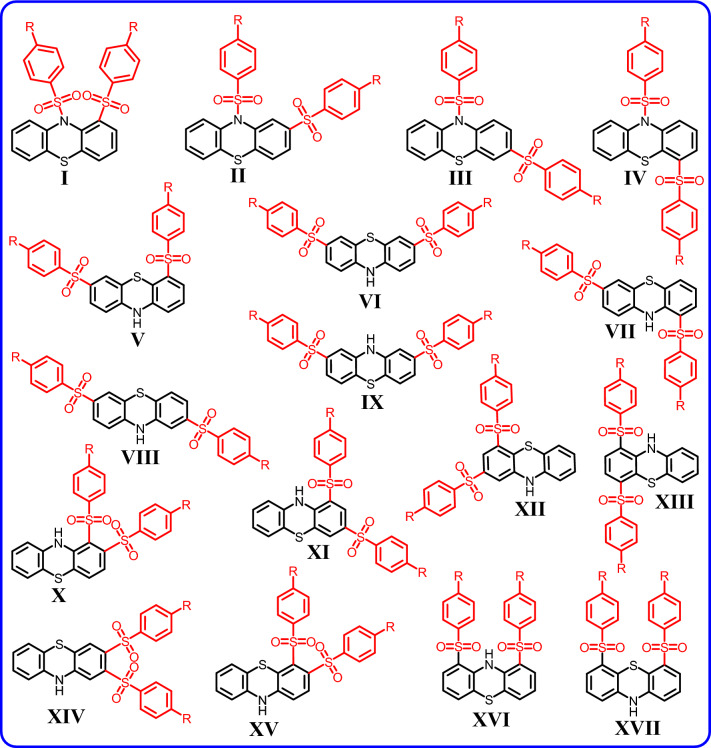
Possible structures in oxidation of **PTZ** in the presence of arylsulfinic acids.

In this part, it is necessary to consider the possibility of formation of different isomers in the oxidation of **PTZ** in the presence of arylsulfinic acids. Figure 8 shows the structures that may be formed in the oxidation of **PTZ** in the presence of arylsulfinic acids. The steric energy calculations show that due to the steric hindrance caused by the presence of two arylsulfinic groups in the *ortho* position to each other, it is not possible to form molecules such as I, X, XIV and XV.

**Figure 9:**
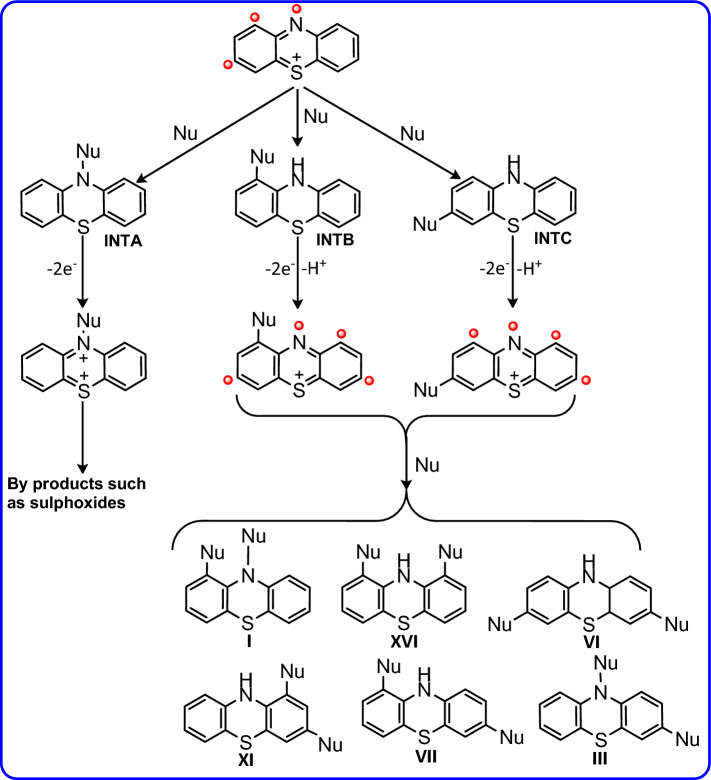
Possible pathways and products after attachment of the first arylsulfinic group.

As discussed earlier, it seems that due to the presence of sulfonium atom in **PTZ**_**OX**_ structure, beside nitrogen atom, C1 and C3 atoms are the most favorable sites for nucleophilic attack. Accordingly, the probability of formation of molecules IX, XII and XVII is very low and therefore they are excluded from the set of possible structures. It seems that the first nucleophilic attack leads to the formation of one of the following intermediates (**INTA**, **INTB** and **INTC**) shown in Fig. 9.

**Figure 10:**
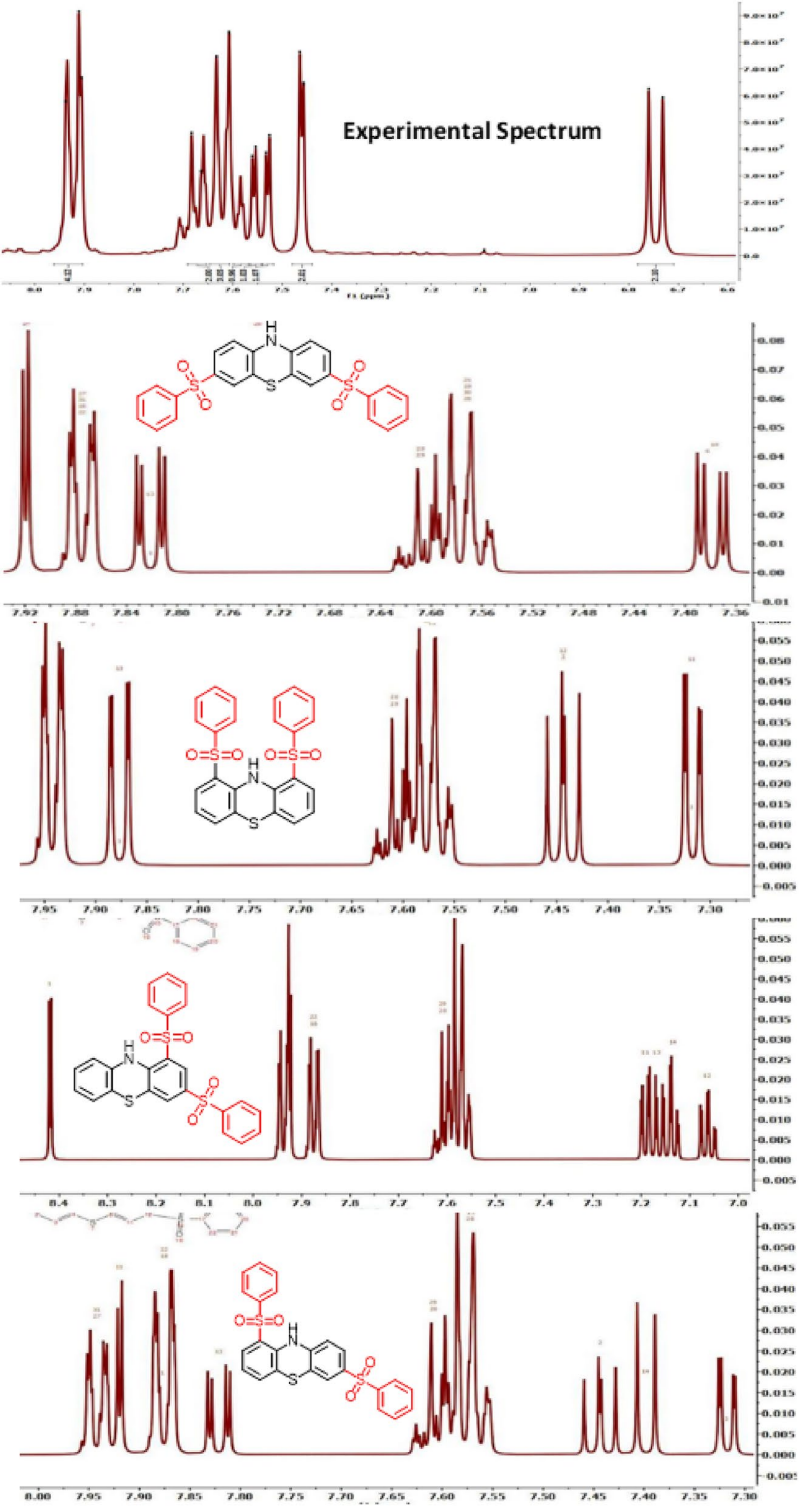
Experimental ^1^H NMR spectrum of **2b** and simulated ^1^H NMR spectra of different compounds.

As discussed, the second oxidation step of the **INTA** leads to the corresponding dication, which is a very unstable compound and does not appear to be capable of conversion to our desired product (molecule III, **2a**). Therefore, we do not consider this pathway possible in the oxidation of **PTZ** in the presence of arylsulfinyl acids. However, the second oxidation step of the intermediates **INTB** and **INTC** leads to the compounds I, III, VI, VII, XI and XVI. By removing compound I for the reasons mentioned earlier, the only remaining compound with the presence of a sulfinic group on the nitrogen atom is compound III (**2a**). By excluding, compounds I and III, the ^1^H NMR spectrum of the product was compared with the simulated ^1^H NMR spectra of compounds VI, VII, XI and XVI. This comparison shows that the experimental spectrum is the most consistent with the simulated spectrum for compound VI (Fig. 10).

### Controlled potential coulometry

**Figure 11:**
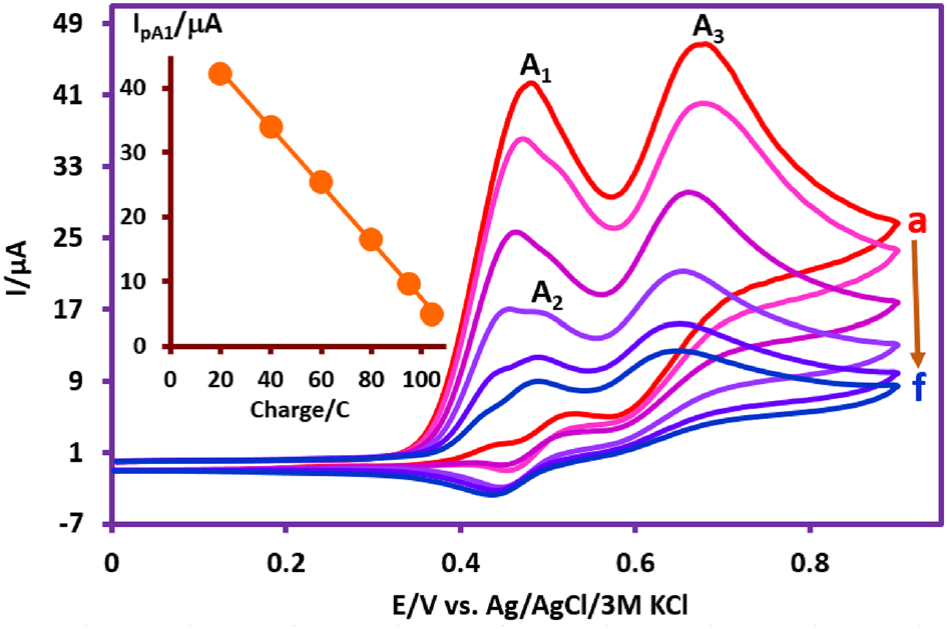
Cyclic voltammograms of 0.25 mmol **PTZ** in the presence of 0.5 mmol **BSA** during controlled-potential coulometry at 0.55 V vs. Ag/AgCl, in phosphate buffer (pH = 2.0, *c* = 0.2 M)/acetonitrile mixture (50/50 v/v). Cyclic voltammograms from a to f are: 20, 40, 60, 80, 95 and 104 C. Scan rate: 100 mV/s. Inset: Variation of A_1_ peak current (*I*_pA1_) vs. charge consumed. Working electrode: glassy carbon electrode. All experiments were performed at room temperature.

Controlled potential coulometry was carried out in a solution containing 0.25 mmol of **PTZ** and 0.5 mmol **BSA** in phosphate buffer (pH 2.0, *c* = 0.2 M/acetonitrile solution mixture (50/50, v/v) at 0.55 V versus Ag/AgCl. In order to better understand what happens during coulometry, cyclic voltammograms of the electrolyzed solution were recorded during coulometry (Fig. 11). These voltammograms at different time intervals show that the peak A_1_ current decreases in the progress of electrolysis. The second change observed in cyclic voltammograms is the appearance of peak A_2_ and its relative increase with the progress of coulometry. Plotting the values of peak A_1_ current versus the amount of electricity consumed in order to determine the number of electrons consumed in this process shows that peak A_1_ disappears with the consumption of about 104 coulombs of electricity (Fig. 11, inset).

According to the electricity consumption and performing the necessary calculations, 4.1 electrons are assigned to each molecule of **PTZ**. This number is slightly higher than 4 due to electrolysis in an undivided cell. The number of electrons obtained in this experiment (*n* = 4) is consistent with that reported in Fig. 4. According to Fig. 4, two electrons are used to oxidize **PTZ** to **PTZ**_**ox**_ (peak A_1_) and the other two electrons are used to oxidize intermediate **INT1** to **INT1**_**ox**_ (peak A_2_). It should be noted that, the lack of increase in *I*_pA3_ and *I*_pC3_ during electrolysis is due to the insolubility of final products in electrolysis solution (see experimental section).

### Constant current synthesis and optimization of effective parameters

**Figure 12:**
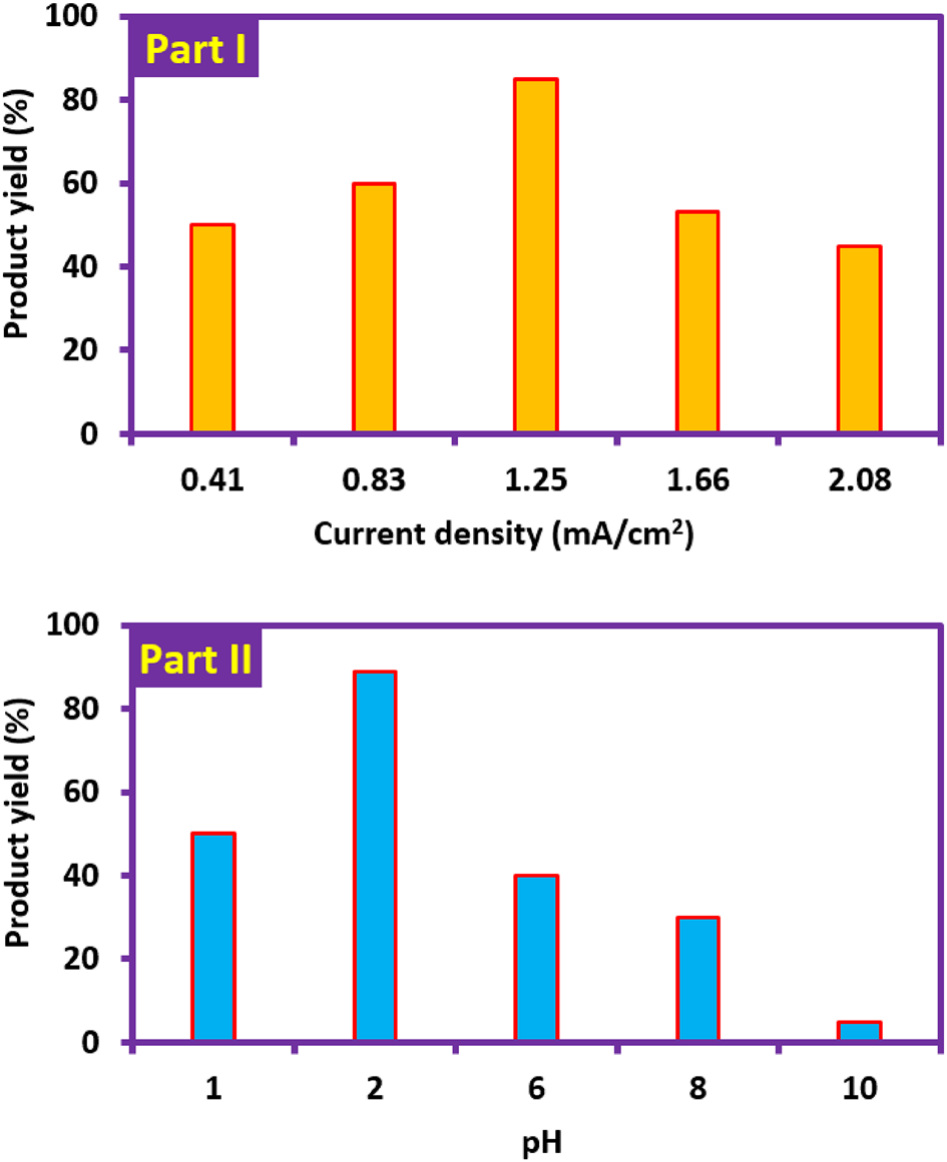
Part I: The effect of current density on the product yield (both **2a** and **2b**). The amounts of **PTZ** and **BSA** 0.25 and 0.5 mmol, respectively. *Q* = 140 C. Solvent: phosphate buffer (pH = 2.0, *c* = 0.2 M)/acetonitrile mixture (50/50 v/v). Anode and cathode material: carbon and stainless steel, respectively. Part II: The effect of solution pH on the yield of product. Applied current density: 1.25 mA/cm^2^. Other conditions are the same as part I. All experiments were performed at room temperature.

In order to facilitate the synthesis of these compounds in a way that can be used by all researchers, in this section, the synthesis of these compounds in constant current mode has been examined and the effective parameters in improving the efficiency and purity of the products, such as applied current density, amount of electricity, solution pH, electrode material and solvent mixture are optimized by one factor at a time method. The current density is one of the most important parameters in electrosynthesis of organic and inorganic compounds, which affects the yield and purity. In this section, the electrochemical synthesis of products (**2a** and **2b**) was investigated at different current densities from 0.41 to 2.08 mA/cm^2^ (Fig. 12, part I) while other parameters are kept constant (see the caption of Fig. 12). The results showed that the highest product yield (89%) was obtained at a current density of 1.25 mA/cm^2^. At current densities less than 1.25 mA/cm^2^, the amount of overvoltage is not sufficient to oxidize **PTZ** and/or intermediates. On the other hand, at current densities higher than the optimal current density (1.25 mA/cm^2^), oxidation of the solvent, supporting electrolyte and/or over-oxidation of the product will reduce the production yield (Fig. 12, part I). To optimize the amount of electricity, the electrochemical synthesis of **2a** and **2b** was investigated at different amount of electricity and at current density of 1.25 mA/cm^2^ while other parameters are kept constant. The results showed that the highest product yield was obtained at *Q* = 140 C. The higher amount of electricity consumed in the constant current method compared to the controlled potential method is due to the inherent difference of the two methods in the selective consumption of electricity.

The effect of solution pH on the yield and purity of products was also investigated (Fig. 12, part II). For this purpose water with different pH values of 1.0, 2.0, 6.0, 8.0 and 10.0/acetonitrile (50/50 v/v) solution mixtures are prepared. Phosphate buffer (0.2 M) was used to prepare solutions with pH values 2.0, 6.0 and 8.0. For pH = 1.0, perchloric acid solution (0.1 M) and for pH = 10.0, carbonate buffer (0.2 M) was used. Other conditions such as current density (1.25 mA/cm^2^) and electricity consumption (140 C) were constant in all experiments. Figure 12, part II shows that the optimum pH is 2.0 for highest product yield. The instability of **PTZ**_**ox**_ in alkaline solutions and participation in side reactions such as dimerization^10,39,40^ and/or hydroxylation^10,41,42^ is the main reason for the low yield of the product in alkaline solutions. On the other hand, the one-electron oxidation of **PTZ** in highly acidic solutions^10^ and the instability of cation radicals along with nucleophile protonation, reduce the product yield in highly acidic solutions.

In this section, the effect of electrode materials on product efficiency is investigated and the results are given in Table 1. As can be seen, the highest product yield was achieved with carbon anode and stainless steel cathode in aqueous phosphate buffer (pH, 2.0, *c* = 0.2M)/acetonitrile mixture (50/50 v/v). The last optimization in this work was performed on the solvent system. The use of organic co-solvent is due to the low solubility of **PTZ** in water. Therefore, solvents such as ethanol and acetonitrile have been used as co-solvents. It should be noted that **PTZ** is easily soluble in acetone. But due to the unexpected reactivity of acetone as a solvent, this compound was not examined as a co-solvent.

**Table 1:** Optimization of electrode material for the synthesis of **2a** and **2b**.


Entry	Anode	Cathode	Yield (%)^a,b^
1	carbon	stainless steel	89
2	stainless steel	stainless steel	20
3	stainless steel	carbon	35
4	titanium	stainless steel	50
5	Ti/β-PbO_2_/CeO_2_^c^	stainless steel	75

^a^The yield reported is the sum of yields of products **2a** and **2b.**

^b^Applied current density: 1.25 mA/cm^2^. Electricity consumption: 140 C. Solvent: phosphate buffer (pH = 2.0, *c* = 0.2 M)/acetonitrile mixture (50/50 v/v).

^c^The electrode was fabricated according to the procedure reported in reference^46^.

The effect of solvent system on product yield is shown in Table 2. As can be seen, the highest product yield was achieved in aqueous phosphate buffer (pH, 2.0, *c* = 0.2 M)/acetonitrile mixture (50/50 v/v). It should be noted that increasing the percentage of acetonitrile in the mixture does not have a significant effect on the yield of the product. In addition, our research has shown that replacing ethanol with acetonitrile in the solvent mixture decreases yield. The decrease may be due to the low solubility of **PTZ** in ethanol. A large increase in the percentage of ethanol in the water/ethanol mixture increases the solubility of **PTZ** (slightly), but on the other hand, it causes a marked decrease in the solubility of the supporting electrolyte. Regarding the solution pH, it should be noted that increasing the pH from 2.0 decreases the yield of **2a** and **2b**. Increasing pH causes deprotonation of **PTZH**^**+**^ and coupling reaction of **PTZ** with **PTZ**_**ox**_ (dimerization reaction)^10^. This reaction competes with the reaction between arylsulfinic acids and **PTZ**_**ox**_. In other words, decreasing the pH causes the protonation of **PTZ** (formation of **PTZH**^**+**^) and suppresses the dimerization reaction. It should be noted that due to the low basicity of arylsulfinate anions (p*K*_a_ < 2.0)^47^, decreasing the pH does not have a significant effect on their nucleophilicity.

**Table 2:** Optimization of the solvent system for the synthesis of **2a** and **2b**.


Entry	Organic co-solvent	Aqueous solution, pH	Yield (%)^a,b^
1	EtOH	phosphate buffer (pH, 2.0, *c* = 0.2 M)	20
2	CH_3_CN	phosphate buffer (pH, 2.0, *c* = 0.2 M)	89
3	–	phosphate buffer (pH, 2.0, *c* = 0.2 M)	no reaction
4	CH_3_CN	phosphate buffer (pH, 6.0, *c* = 0.2 M)	40

^a^The yield reported is the sum of yields of products **2a** and **2b**.

^b^Applied current density: 1.25 mA/cm^2^. Electricity consumption: 140 C. Solvent: phosphate buffer (pH = 2.0 or 6.0, *c* = 0.2 M)/organic solvent mixture (50/50 v/v). Anode: carbon. Cathode: stainless steel.

## Conclusion

In this study, the electrochemical synthesis of five new phenothiazine derivatives (bis(phenylsulfonyl)-10*H*-phenothiazine derivatives) in water/acetonitrile mixture was carried out in a one-pot process through the electrochemical generation of phenothiazin-5-ium (**PTZ**_**ox**_) in the presence of arylsulfinic acids. Our results show that two different types of products (bis(phenylsulfonyl)-10*H*-phenothiazine derivatives) are formed during the electrolysis: (1) Phenothiazine-sulfonamide-sulfone derivatives. In these products, one arylsulfinic group is attached to the nitrogen atom and the other group is attached to the carbon atom. (2) Phenothiazine-disulfone derivatives. In these products, both arylsulfinic groups are attached to carbon atoms. In addition to the controlled potential method, the synthesis of these compounds by performing electrolysis in constant current mode has also been successful. In this research, a mechanism for the oxidation of PTZ in the presence of arylsulfinic acids was also proposed based on the data obtained from cyclic voltammetric, chronoamperometric and controlled-potential coulometric studies along with the structure of the synthesized products. This mechanism is depicted in Fig. 4. According to Fig. 4, **PTZ** is converted to the final product (bis(phenylsulfonyl)-10*H*-phenothiazine derivatives) through the *ECEC* mechanism. The insolubility of the final product in the electrolysis solution is one of the factors that prevent the re-oxidation of the final product. Finally, we hope that the synergistic effect of the groups added to the phenothiazine molecule will intensify the medicinal properties and/or reduce the side effects of the synthesized molecules.

## Data Availability

All data generated or analyzed during this study are included in this published article.
